# Neurocircuit Assays for Seizures in Epilepsy Mutants of *Drosophila*

**DOI:** 10.3791/1121

**Published:** 2009-04-15

**Authors:** Iris C. Howlett, Mark A. Tanouye

**Affiliations:** Department of Molecular and Cell Biology, University of California, Berkeley; Department of Environmental Science, Policy Management, University of California, Berkeley

## Abstract

*Drosophila melanogaster* is a useful tool for studying seizure like activity. A variety of mutants in which seizures can be induced through either physical shock or electrical stimulation is available for study of various aspects of seizure activity and behavior. All flies, including wild-type, will undergo seizure-like activity if stimulated at a high enough voltage. Seizure like activity is an all-or-nothing response and each genotype has a specific seizure threshold. The seizure threshold of a specific genotype of fly can be altered either by treatment with a drug or by genetic suppression or enhancement. The threshold is easily measured by electrophysiology. Seizure-like activity can be induced via high frequency electrical stimulation delivered directly to the brain and recorded through the dorsal longitudinal muscles (DLMs) in the thorax. The DLMs are innervated by part of the giant fiber system. Starting with low voltage, high frequency stimulation, and subsequently raising the voltage in small increments, the seizure threshold for a single fly can be measured.

**Figure:**
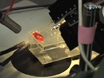


## Protocol

### Specimen Preparation:

The night before the experiment, male flies of the desired genotype are anesthetized and placed in a vial with a soft nylon plug. A cotton ball would also work. Following anesthetization, the seizure threshold is altered, so the flies are best left for at least 6 hours, though over night is ideal.

On the day of the experiment, the flies are mounted one at a time to glass slides with a layer of dental wax on one side.

A single fly is picked up by the head using a 23 gauge needle (with the sharp tip cut off) attached to a vacuum line.

The fly is then affixed to the wax on the slide by pushing the legs and wings into the wax.

Once the fly is immobilized, wax is pushed up around the neck and eyes to fully immobilize the head.

### Electrophysiology:

The fly, which is now mounted and ready to go is placed in the electrophysiology set up.

The electrophysiology set up consists of three sets of micromanipulators, each with one to two sharpened tungsten electrodes attached. Voltage is provided by a stimulator that is connected to an isolation unit attached to the stimulating electrodes. The recording electrode is connected to a pre-amp which in turn is attached to a digital storage oscilloscope. While it is not essential, it is useful to have a speaker hooked up to the oscilloscope so that responses can be made audible.

A ground electrode is inserted into the abdomen of the fly.

The recording electrode is inserted into one of the Dorsal Longitudinal Muscles in the thorax of the fly.

The stimulating electrodes are inserted into the head. One to the side of each eye.

The stimulator should have the following settings to start: Continuous pulse interval with 2 seconds between each pulse. There should be no pulse delay. The pulse width should be 0.2ms.

The oscilloscope should have the storage feature turned on and have the following settings: Time/DIV should be set to 1ms/DIV. The Volts/DIV should be set at either 0.1V/DIV or 50 mV/DIV.

The giant fiber threshold is determined by delivering single pulses and noting the minimal voltage required to get a response. This voltage is typically around 2 V.

Once the giant fiber threshold has been determined, stop pulsing the fly and change the settings on the oscilloscope to: 0.2 S/DIV

The settings on the stimulator should be changed to: The pulse interval should be set to single. The train should be turned on and set to 3 seconds. The waveform parameters are: 0.5 ms pulses at 200Hz for 300 ms. The voltage is changed to the desired setting and a high-frequency train of pulses is delivered to the fly’s brain.

If a seizure is not induced, the oscilloscope trace will show a flat line after the train. If the settings are quickly set back to the starting settings, the single pulses will cause a response in the giant fiber system.

If a seizure is induced, the oscilloscope will show the seizure-like activity after the train. If the settings are quickly switched back to the original settings, the single pulses will fail to activate a response in the giant fiber system.

The flies have a refractory period after a high-frequency stimulus wavetrain in which seizure like activity cannot be induced. If no seizure occurs the flies are refractory for approximately 7 minutes. If a seizure is induced, the flies have a refractory period of approximately 17 minutes.

When determining the threshold, additional high-frequency stimuli can be delivered to the same fly (after the appropriate time for the refractory period) until the threshold is determined or until the giant fiber response becomes too weak to see.

After the experiment is completed, the fly is removed from the electrophysiology set up, released from the wax, and disposed of.

## Discussion

This video has demonstrated the basic steps in determining the seizure threshold of an individual *Drosophila* fly. Instead of using the digital oscilloscope, a computerized digitizer may also be used. This technique is useful for determine the effects of a drug or genetic alteration on the seizure threshold of a fly.

